# Evaluating the Efficiency of Baited BioFlyTrap P38T, H-Trap, and Nzi and Vavoua Traps Towards Biting Flies of Glossinidae, Tabanidae and Muscidae (Stomoxyini) Families in Northeastern KwaZulu-Natal

**DOI:** 10.3390/insects17090912

**Published:** 2026-09-01

**Authors:** Moeti O. Taioe, Serero A. Modise, Solomon N. B. Boikanyo, Mamohale Chaisi, Geoffrey Gimonneau, Johan Esterhuizen, Marc Desquesnes

**Affiliations:** 1 Epidemiology, Parasites & Vectors, Agricultural Research Council-Onderstepoort Veterinary Research (ARC-OVR), Onderstepoort 0110, South Africa; boikanyos@arc.agric.za (S.N.B.B.); jsoliterra@gmail.com (J.E.); 2 Unit for Environmental Sciences and Management, North-West University, Potchefstroom 2520, South Africa; 3 Foundational Biodiversity Science, South African National Biodiversity Institute, Pretoria 0001, South Africa; sereromodise@gmail.com (S.A.M.); m.chaisi@sanbi.org.za (M.C.); 4 Department of Veterinary Tropical Diseases, Faculty of Veterinary Science, University of Pretoria, Onderstepoort 0110, South Africa; 5 INTERTRYP, University of Montpellier, Cirad, IRD, F-34398 Montpellier, France; geoffrey.gimonneau@cirad.fr; 6 Centre de Coopération Internationale en Recherche Agronomique pour le Développement, Unité Mixte de Recherche, INTERTRYP, F-34398 Montpellier, France; marc.desquesnes@cirad.fr; 7 National Veterinary School of Toulouse, National Research Institute for Agriculture, Food and Environment, Host-Pathogen Interactions Laboratory, University of Toulouse, 31062 Toulouse, France

**Keywords:** biting flies, vector surveillance, Latin Square Design, P38T BioFlyTrap

## Abstract

In efforts to survey biting flies, there is a need for a multi-species trap that can capture diverse families of hematophagous flies, thus functioning as a more robust and representative trapping method. The current study evaluated the efficiency of the baited P38T BioFlyTrap with other standard traps, namely the H-trap used in South Africa, the Vavoua (West/Central Africa), and the Nzi (East Africa), in informing decision-making to undertake enhanced surveillance and control interventions against biting flies in South Africa. A comparative study was done using a 4 × 4 Latin Square Design (LSD) approach over a period of 16 days in northeastern KwaZulu-Natal. The H-trap was most efficient for Glossinidae, while the Nzi trap caught the most diverse species dominated by Tabanidae. The P38T BioFlyTrap showed moderate performance across all categories (Glossinidae, Tabanidae, Muscidae (Stomoxyini)) of biting flies. The Vavoua trap was the least effective trap for Glossinidae and Tabanidae, but was relatively effective for Stomoxyini. In this environment, baited P38T BioFlyTrap would be useful for broader spectrum surveillance, while baited H-trap and Nzi remain specifically more performant, respectively for Glossinidae and Tabanidae.

## Simple Summary

Sampling of large biting flies (tsetse flies, horse flies and stable flies) requires a variety of trapping devices as these insects respond differently to the current trapping tools developed. There is therefore a need to have a multi-species trap that can capture diverse families of hematophagous insects and trap enough representatives of those families within a short period. The current study used a 4 × 4 Latin Square Design (LSD) to demonstrate the efficiency of the baited P38T BioFlyTrap and other traps (H-trap, Nzi and Vavoua) in capturing large biting flies. The Nzi trap captured most biting flies, the H-trap was more efficient for tsetse flies and the Vavoua trap was least efficient in capturing tsetse and horse flies. The P38T BioFlyTrap demonstrated moderate performance for all biting insects (tsetse flies, horse flies and stable flies) and it could be recommended for a wide range of large biting fly investigations, while the other traps (H-trap, Nzi and Vavoua) are better used for targeted insect surveillance.

## 1. Introduction

Animal African trypanosomosis (AAT) is a protozoan disease that has a detrimental effect on agricultural productivity and livelihoods in endemic areas of sub-Saharan Africa [1]. Infected animals display a variety of symptoms ranging from intermittent fever, anorexia, anemia, severe muscle wasting and emaciation, reduced milk yields, abortions, and death if untreated, resulting in significant economic loss and poverty [2]. Tsetse flies (Glossinidae: *Glossina* spp.) are biological vectors of AAT, while stable flies (*Stomoxys* spp.) and clegs/horse flies (Tabanidae) have been implicated in the mechanical transmission of AAT [3,4,5]. Control of AAT and its vector flies is mainly through an integrated approach focusing on the reduction or possible elimination of tsetse fly populations [6] and treating infected livestock with trypanocidal drugs [7]. However, due to long-term usage (some of the trypanocidal drugs been in use for more than 70 years), poor farming management practices and resistance to trypanocidal drugs, which has been reported in several countries [8], alternative methods such as vector control strategies are recommended alongside drug treatment [9].

Various vector control and monitoring methods have been developed, tested, and implemented in several countries based on the biology, ecology, and habitat preferences of insect vectors to reduce the burden imposed by the disease [10]. Early tsetse control efforts involved clearing of vegetation to destroy adult tsetse resting and breeding sites, extensive hunting of wild animals to remove possible blood sources for the flies, as well as aerial and ground spraying with residual insecticides [11]. However, due to their limited effect on the target insect vector, high costs, labor demands, and subsequent environmental consequences, these techniques were considered ineffective and undesirable [10,12,13]. Recently, more selective and environmentally friendly methods have been used to control tsetse flies, and these include the use of insecticide-treated screens (targets) to attract and kill the tsetse vectors and insecticide applications on host animals, either as pour-on applications or neck collars [14,15], as well as repellents and the sterile insect technique [10,16], of which majority have shown promising outcomes in reducing tsetse populations. Insect trap designs that are used more for population surveillance than control of the vectors like tsetse and other large biting flies of Tabanidae (horse flies) as well as Stomoxyini (stable flies) have been developed to catch different species of tsetse and other large biting flies in different geographical locations [17,18]. These make use of phthalogen-blue, black and white cloths for visual stimuli [19,20], and attractants (carbon dioxide at 2 L/min, acetone at 5 mg/h, butanone at 0.5 mg/h, 1-octen-3-ol (octenol) at 0.05 mg/h, 4-methyl phenol at 0.05 mg/h, and 3-n-propyl phenol at 0.005 mg/h) for olfactory stimuli [16,21].

There are 31 described species and subspecies of tsetse flies distributed across 34 sub-Saharan African countries, divided into the Savannah Group (*Morsitans*), Riverine Group (*Palpalis*), and the Forest Group (*Fusca*), that respond differently to various trapping devices and attractants [22]. The current traps used are according to the target tsetse species, whereby the NGU/NGURMAN trap [23] and the Nzi trap [20] are mostly used in East Africa for the Savannah group of flies, while the biconical trap [24], the Vavoua trap [25], and the pyramidal trap [26] are utilized mostly in Central and Western African countries to control the Riverine group and some species from the Savannah group [13,27]. Lastly, there is the Epsilon trap [28] used in southern Africa against the Savannah group and the H-trap [19] used for the Forest and Savannah groups of flies in South Africa [17].

In South Africa, only two species of tsetse flies occur, namely *Glossina brevipalpis* and *G*. *austeni*, confined to the northeastern parts of KwaZulu-Natal Province (KZN), covering approximately 16,000 km^2^ [19,29,30]. These two tsetse species have been reported to be responsible for AAT in KZN [31,32,33], and Taioe et al. [34] implicated some Tabanidae in the disease transmission from the same area. Currently in South Africa, odor-baited H-traps are used for the monitoring and surveillance of large biting flies such as horse, stable and tsetse flies [35]. However, there is a need for a multi-species trap that can capture diverse families of hematophagous insects, thus functioning as a more robust and representative trapping method. To develop sustainable vector control tools, this study evaluated the efficiency of a novel, biodegradable, odor-baited BioFlyTrap (P38T) prototype [36,37] against three standard traps, namely, the H-trap (southern Africa), the Vavoua trap (West/Central Africa), and the Nzi trap (East Africa). Performance was assessed based on multi-species catches of hematophagous dipterans, specifically targeting Glossinidae, Tabanidae, and Stomoxyini. This initiative is to inform decision-making to undertake enhanced surveillance and control interventions against large biting flies in South Africa. Additionally, this study forms part of the 2nd Pillar (Innovative control tools) of the COMBAT project that seeks to develop environmentally friendly vector control tools capable of targeting both tsetse and mechanical vectors [38].

## 2. Materials and Methods

### 2.1. Study Area

**Figure 1:**
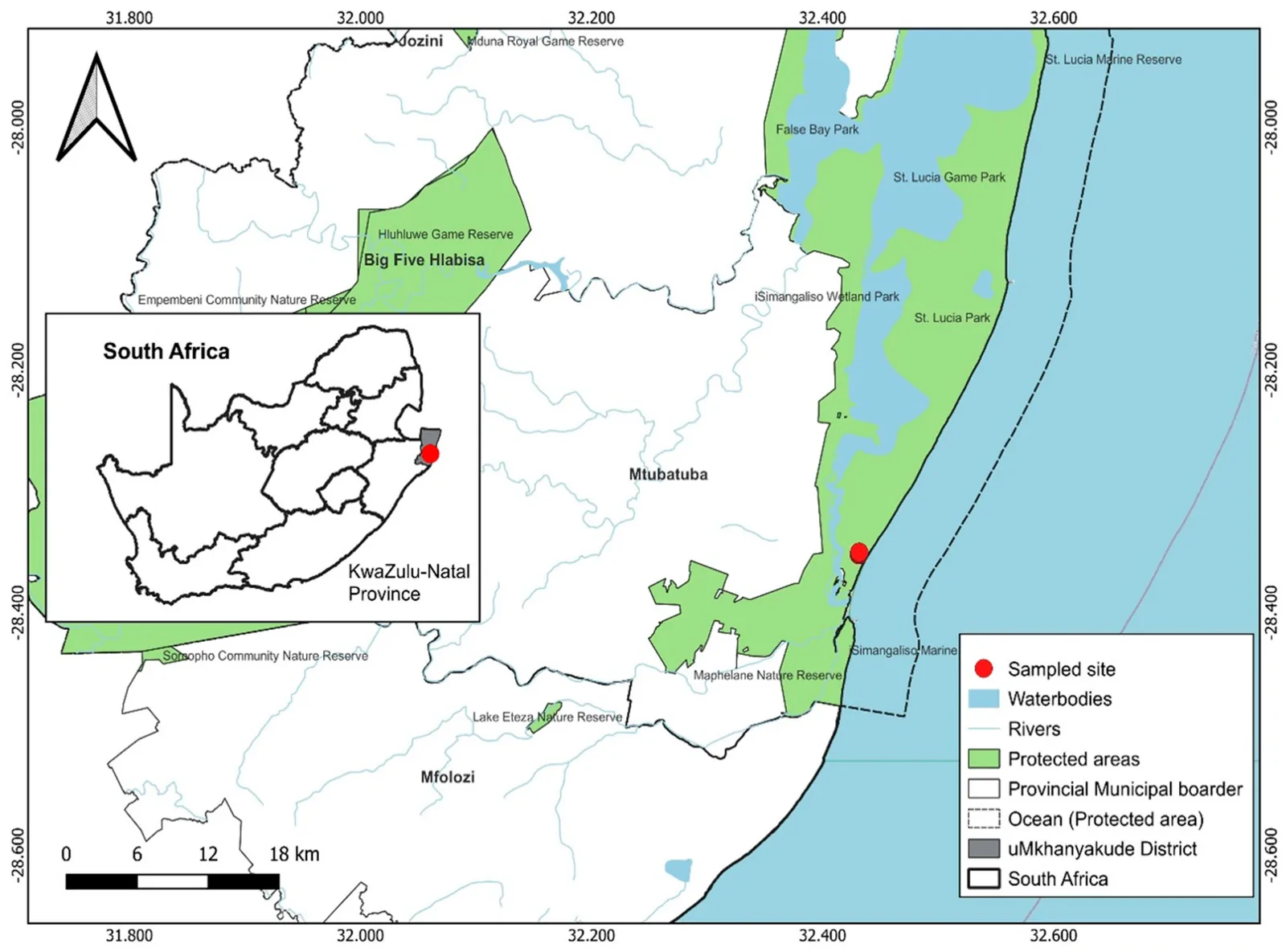
Location of the experimental site where the Latin Square rotations were conducted in St Lucia, northeastern KwaZulu−Natal Province, South Africa.

The experiments were conducted at the Bhangazi Horse Safaris [39] (28°21′10.59″ S, 32°25′51.46″ E) (Figure 1), which is the sole concessionaire for horse safaris from St Lucia in the iSimangaliso Wetland Park [40]—South Africa’s first UNESCO World Heritage Site that falls under the Indian Ocean Coastal Belt vegetation biome dominated by high dunes and a mosaic of mangrove, sandy, savannah and swamp forests [41]. Sampling was conducted during late-spring and early-summer months which are composed of high humidity, frost-free, and subtropical climatic conditions with an average maximum temperature of 27–35 °C, lowest at 17–20 °C and rainfall ranging between 91 and 110 mm precipitation [41].

### 2.2. Trapping Systems

**Figure 2:**
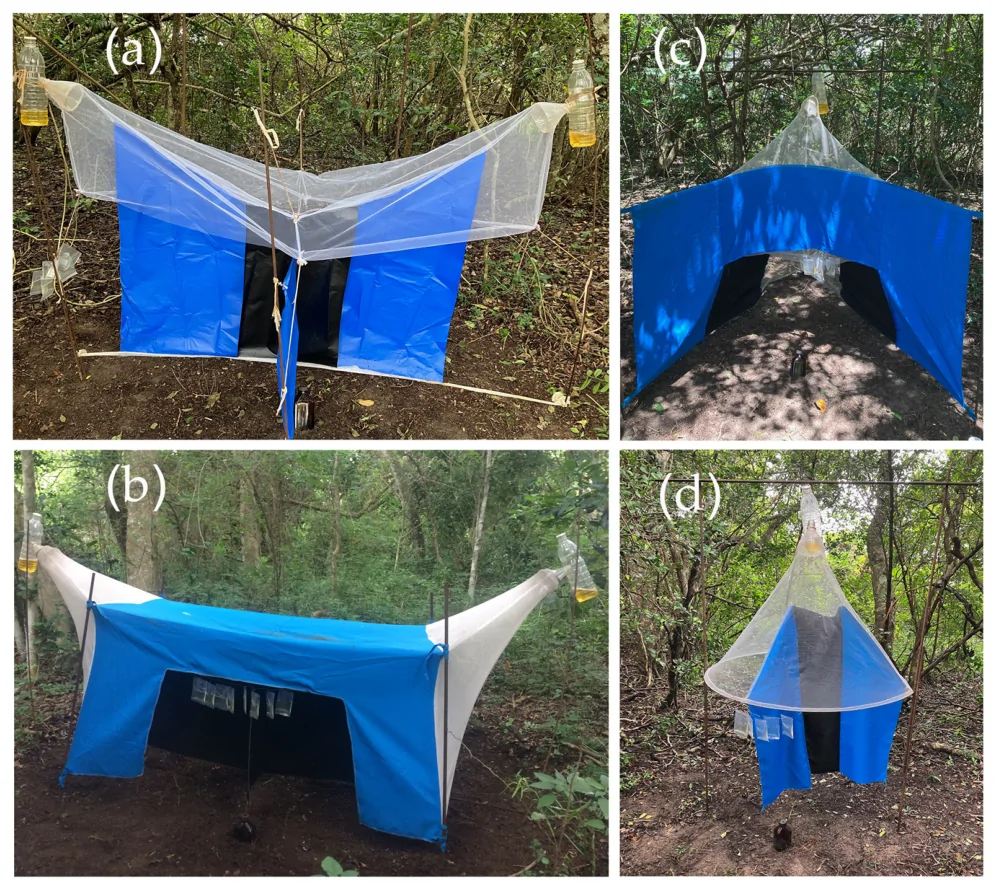
Traps used for the Latin Square experiments included (**a**) P38T BioFlyTrap; (**b**) H-trap; (**c**) Nzi; and (**d**)-Vavoua trap.

The P38T BioFlyTrap (Figure 2a) was designed by CIRAD and ENVT in collaboration with private partners using environmentally friendly biodegradable material (Poly lactic acid) that is not treated with any insecticide [36,37]. The trap design was made with the following terms of reference: universal biting fly trap, made of biodegradable material, suitable for industrial production, and inexpensive. The blue and black parts of the trap are made from biodegradable plastic (the blue plastic reflectance being close to that of phthalogen-blue-stained fabrics), while the white part is from a thin mosquito net (non-biodegradable in this prototype, since a biodegradable transparent mosquito net is not yet available), and plastic bottles are used for collection of trapped insects.

The subsequent traps, considered as reference traps were procured from Vestergaard Frandsen (Vestergaard Frandsen SA Proprietary Ltd., Rivonia, Johannesburg, South Africa) to ensure that the material and colors are uniform for visual attraction.

The H-trap, a horizontal trap (Figure 2b), assumes a rectangular shape, and consists of a blue cloth outer box (100 cm × 65 cm × 65 cm) with two opposite side entrances (40 cm × 45 cm), an inner black cloth X-target (which acts as a baffle attached to the inner center of the roof), and two horizontal cones of white mosquito netting extending laterally from ends of the trap in opposite directions, where an apex of each cone is fitted to a top third of polythene bottle which is also fitted to a second bottle that is used as a collecting device. The trap is held upright by fastening the four corners to rigid metal poles (2 m high) and two cones-collector bottles suspended from two flexible steel rods (1.4 m high) to stand erect on the ground [19].

The Nzi trap (Figure 2c) has equilateral triangular-shaped sides (90 cm each side) flanked by two blue wings (100 cm × 50 cm) at the front. The sides are half black (100 cm × 50 cm) towards the front and half netting (100 cm × 100 cm) towards the back, with a trapezoidal netting (100 cm × 50 cm) shelf extending horizontally from the bottom to the front, then to the midpoint of the sides to make entrances for flying through to the back; the cone is a tetrahedron netting (100 cm × 100 cm) which has a top apex tip that is fitted with a collecting bottle cone The trap is held upright by fastening the three corners to rigid metal poles (2 m high) and a cone-collector bottle suspended from one flexible steel rod (2.5 m high) stands erected from the rare back on the ground [20].

The Vavoua trap (Figure 2d) has a monoconical shape, with an upper cone of polyamide mosquito net (apex angle of 56 degrees, a radius of 78 cm and slant height of 85 cm) over heading three screens, and is composed of a blue external part (cotton/polyester, trapezium-like, 76 cm × 48 cm × 27 cm) and a black internal part (polyamide, 76 cm × 28 cm) with a blue/black ratio equal to 2, and has a cone apex with a circular galvanized wire connected to collecting bottle cone at the top. The trap stands on a 1.5 m steel rod trap sheet about 30 cm to 40 cm from the ground [25].

For olfactory stimuli, all 4 (P38T BioFlyTrap, H-trap, Nzi and Vavoua) traps were baited with 8 semi-permeable polyethylene sachets composed of 1:8 mixtures of 1-octen-3-ol and 4-methylphenol (p-cresol), dispensed at release rates of ca. 4.4 mg/h for octenol and ca. 7.6 mg/h for methylphenol per sachet, and acetone dispensed from a 300 mL glass bottle with a 6 mm hole in the cap at the release rate of ca. 350 mg/h [19,29]. This was done to ensure that flies are attracted to the traps because the tsetse habitat in KZN is composed mostly of dense vegetation.

Lure sachets were placed inside the H-traps according to standard protocol [19]. For the remaining trap designs, which lacked standard placement guidelines, sachets were secured at the entrance of the Nzi traps and along the outer borders of both the Vavoua and P38T BioFlyTrap; these variations in positioning were accounted for during data analysis.

### 2.3. Experimental Design

To evaluate the efficiency of the baited P38T BioFlyTrap against reference traps (H-trap, Nzi, and Vavoua) (Figure 2a–d), a comparative study was done using a 4 × 4 Latin Square Design (LSD) approach [42,43] over a period of 16 days between the 6th and the 22nd of November 2025 when biting flies are most active (early summer). This method has been demonstrated as the most appropriate design for conducting insect trapping experiments [42]. Traps were positioned at least 100 m apart and rotated daily between 09:00 a.m. and 12:00 p.m. Collected fly catches were subsequently counted and categorized by morphospecies. Notably, there was significant rainfall on 8 November 2025 that compromised the traps; therefore, data from this date were omitted from the dataset. To maintain consistency, the traps were re-installed in their original positions, and the daily rotations resumed on the following day, 9 November. Collection bottles were half-filled with 70% ethanol and a 10% Savlon solution (a mixture of double distilled water mixed with commercial Savlon Antiseptic Liquid (Kenvue SA Proprietary Limited, Cape Town, South Africa) that contains 3.0% *w*/*v* Cetrimide and 0.3% *w*/*v* Chlorhexidine Gluconate) to preserve collected specimens, prevent predator insects from eating collected specimens, and prevent fecal DNA cross-contamination among collected insects.

### 2.4. Statistical Analysis

The effectiveness (attractiveness) of the trap types was assessed using a Generalized Linear Mixed Model (GLMM) with a negative binomial distribution (variance = μ + μ^2^/θ), using the total number of insects captured as the response variable. Trap type was included as a fixed effect, while trapping site and trapping date were included as random effects to account for spatial and temporal variability inherent to the Latin Square Design. The significance of the fixed effect (trap type) was evaluated using a Wald chi-squared test (Type III). Pairwise comparisons between trap types were performed using estimated marginal means (emmeans package) with Tukey adjustment; compact letter display was generated using Sidak adjustment as implemented in the emmeans package. Model fit and assumptions were verified using simulated quantile residuals (DHARMa package), including tests for overdispersion and zero-inflation. The daily flies catch rates were determined as absolute number of flies/trap/day to monitor daily performance. Trap catch index (*TCI*) was used to determine relative trap efficiency as a proportion of each morphospecies over the total catch across all tested trap designs and rotation days (Equation (1)).

(1) $$TCI=(\frac{\sum x_{ij}}{N})$$

Equation (1): Trap catch index (*TCI*), *x* is relative abundance count of *i*th morphospecies caught by *j*th trap, x*_ij_* is sum of counts of *i*th morphospecies in all traps, and *N* is grand total of flies caught across all traps and all days.

The species surrogates between traps were compared using log (x + 1) transformation abundance to estimate species accumulation (random), heterogeneity, and community composition using non-metric multidimensional scaling ordination (NMDS, by Bray–Curtis). PERMANOVA was used to test difference in community composition (Bray–Curtis dissimilarity, 999 permutations). All statistical analyses were performed in R version 4.3.2 using the glmmTMB, emmeans, DHARMa and vegan packages at a 5% significance level.

## 3. Results

### 3.1. Trapping Summary of Biting Insects per Family

A total of 1439 biting flies from the families Tabanidae, Muscidae, and Glossinidae were collected during 16 daily trap rotations. The Nzi trap caught the most biting insects, contributing 40.65% of total catches, followed by the H-trap and P38T BioFlyTrap at 31.48% and 16.82%, and the least was the Vavoua trap at 11.05% of catches (Figure 1). The Wald χ^2^ (df = 3) = 34.459 (*p* < 0.05) indicated that all trap types capture varied quantity of biting flies. However, Tukey post test indicated significant differences between Nzi and Vavoua trap (*Z* = 5.425, *p* < 0.001), H and Vavoua trap (*Z* = 5.021, *p* < 0.001) as well as P38T and Vavoua trap (*Z* = 2.809, *p* < 0.05) using pairwise comparisons (Table 1). The Nzi trap demonstrated the highest daily catch rate of 36.56 flies/trap/day, followed by the H-trap with 28.31 flies/trap/day and the P38T BioFlyTrap with 15.13 flies/trap/day, as well as the Vavoua trap that yielded the lowest daily catch rate of 9.938 flies/trap/day (Table 2). Random effects of site and date had no significant difference between trap catches using the Levene homogeneity test comparison of variance.

**Figure 3:**
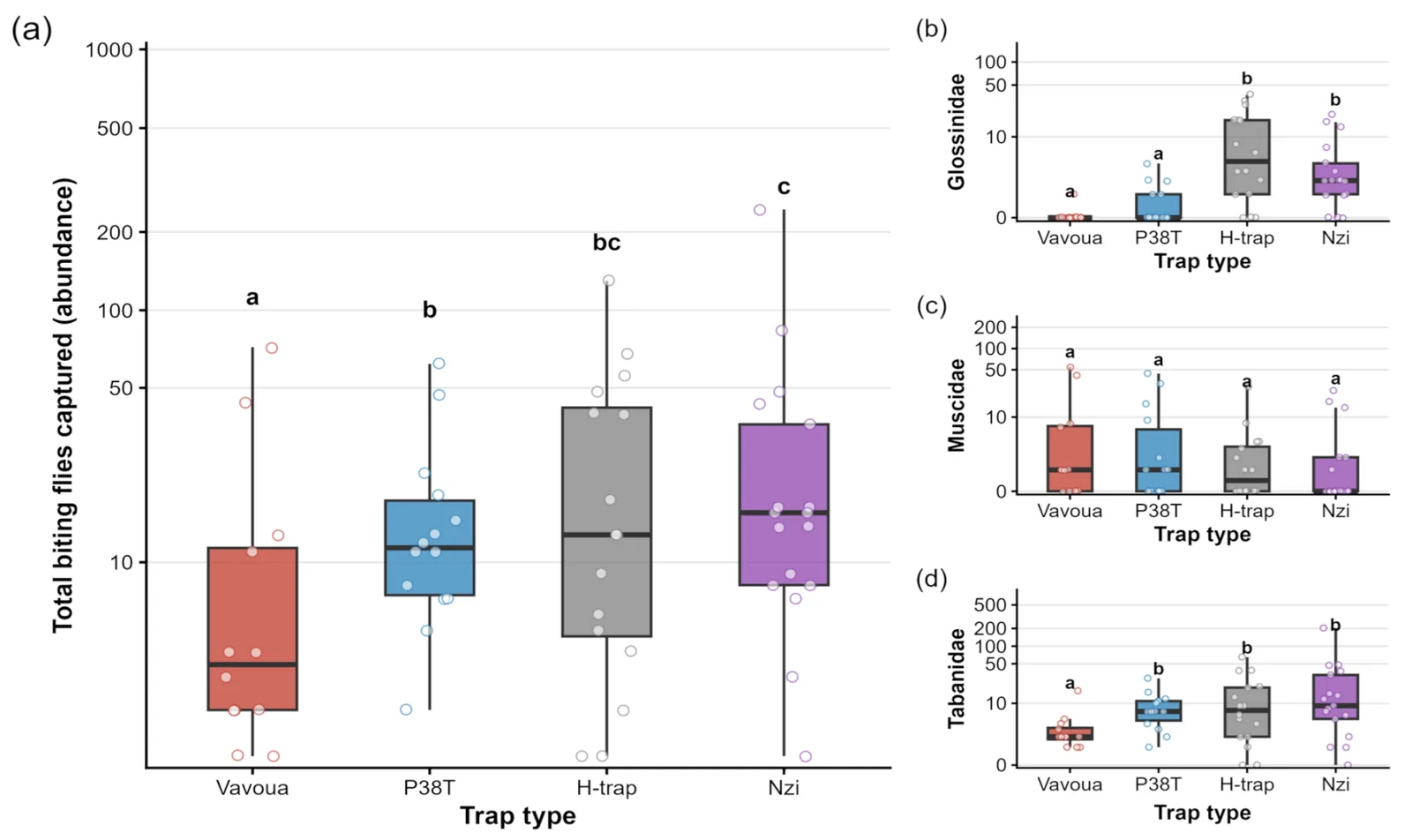
Comparative trap catch for biting flies using total catch (**a**), family comparative catch Glossinidae (**b**), Muscidae (**c**) and Tabanidae (**d**) by different traps over the 16-day sampling period. (box plot bars with same letters indicate no significant difference).

Biting fly families also demonstrated specific affinities for particular trap types. A highly significant difference was observed between the H-trap and other trap models (*p* < 0.01) regarding the number of Glossinidae captured (Figure 3b). Specifically, the H-trap yielding a high trap catch index value of 0.659 for Glossinidae, the Nzi trap demonstrated intermediate performance with a *TCI* = 0.298, while lower performance was shown for P38T BioFlyTrap (*TCI* = 0.039) and the Vavoua trap (*TCI* = 0.004) respectively.

No statistically significant differences were observed among the evaluated trap types regarding the capture rates of *Stomoxys calcitrans*, *S. niger niger*, and *Haematobia* spp. (Figure 3c). Both Vavoua (*TCI* = 0.347) and P38T (*TCI* = 0.317) recoded higher trap catch index, and there were lower relative efficiency for the Nzi (*TCI* = 0.183) and H-trap (*TCI* = 0.153) respectively.

The Nzi trap yielded the highest trap catch index for Tabanidae with *TCI* = 0528, while H-trap (*TCI* = 0.274) and the P38T BioFlyTrap (*TCI* = 0.149) exhibited intermediate relative catch efficiency, which did not differ significantly from each other (Figure 3d). Conversely, the Vavoua trap (*TCI* = 0.050) demonstrated the lowest efficiency. Pairwise comparisons revealed highly significant differences between the Vavoua trap and all other evaluated trap types (*p* < 0.01). The captured Tabanidae fauna comprised representatives from seven genera: *Ancala*, *Chrysops*, *Haematopota*, *Hybomitra*, *Philoliche*, *Tabanocella*, and *Tabanus* spp.

### 3.2. Species Accumulation per Trap

**Figure 4:**
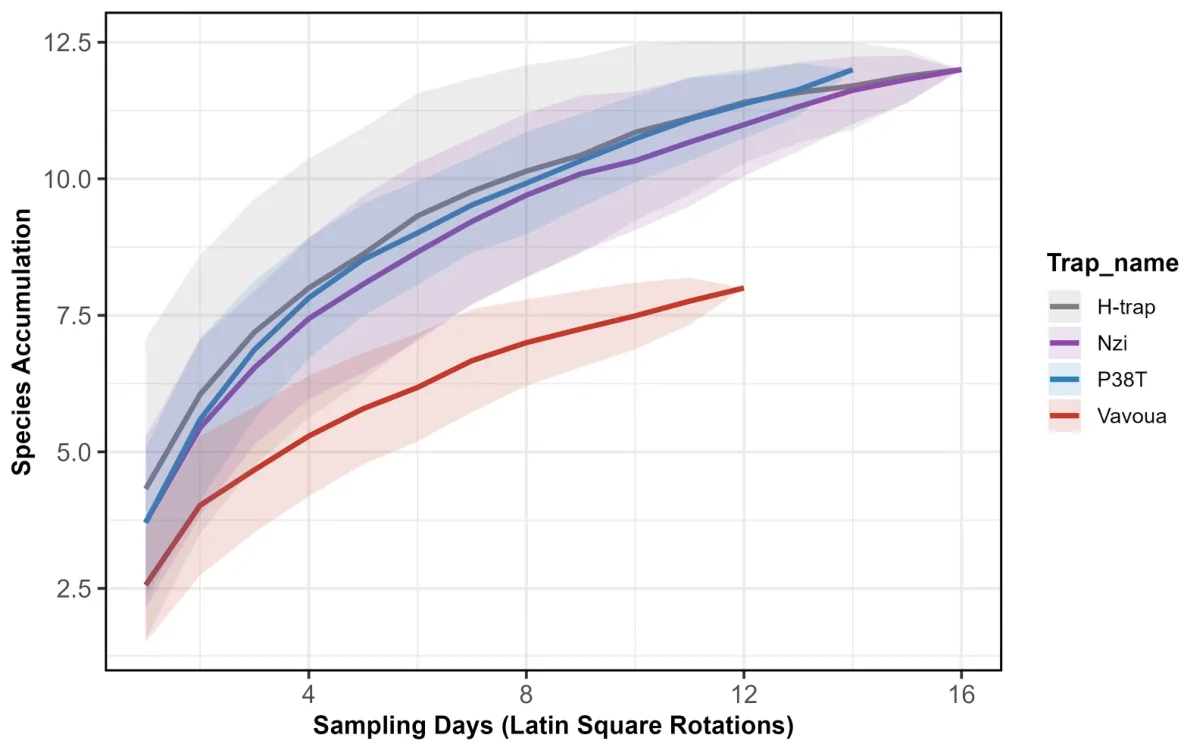
Species accumulation curve between the H-trap, Nzi, P38T BioFlyTrap and Vavoua used for the collection of biting flies over a 16-day sampling period.

In an effort to assess if sufficient sampling was conducted to completeness, a species accumulation curve was plotted to represent the cumulative number of unique species detected against the increasing number of trap events. The H-trap, Nzi, and P38T BioFlyTrap had steep slopes over 16 days of sampling, indicating efficiency in the initial trapping period by accumulating 12 biting fly species each, whilst the Vavoua trap was least efficient and accumulated only eight unique species over the same sampling period (Figure 4). The H-trap and P38T BioFlyTrap both had steep inclines in the first few days, indicating that they are more efficient in capturing a wide variety of species in a short time, and the Vavoua was the least efficient. All four trap type curves did not reach a plateau, suggesting that with prolonged sampling rotations, new biting fly species could be added.

### 3.3. Species Heterogeneity (Shannon) Index and Community Assemblages by NMDS

**Figure 5:**
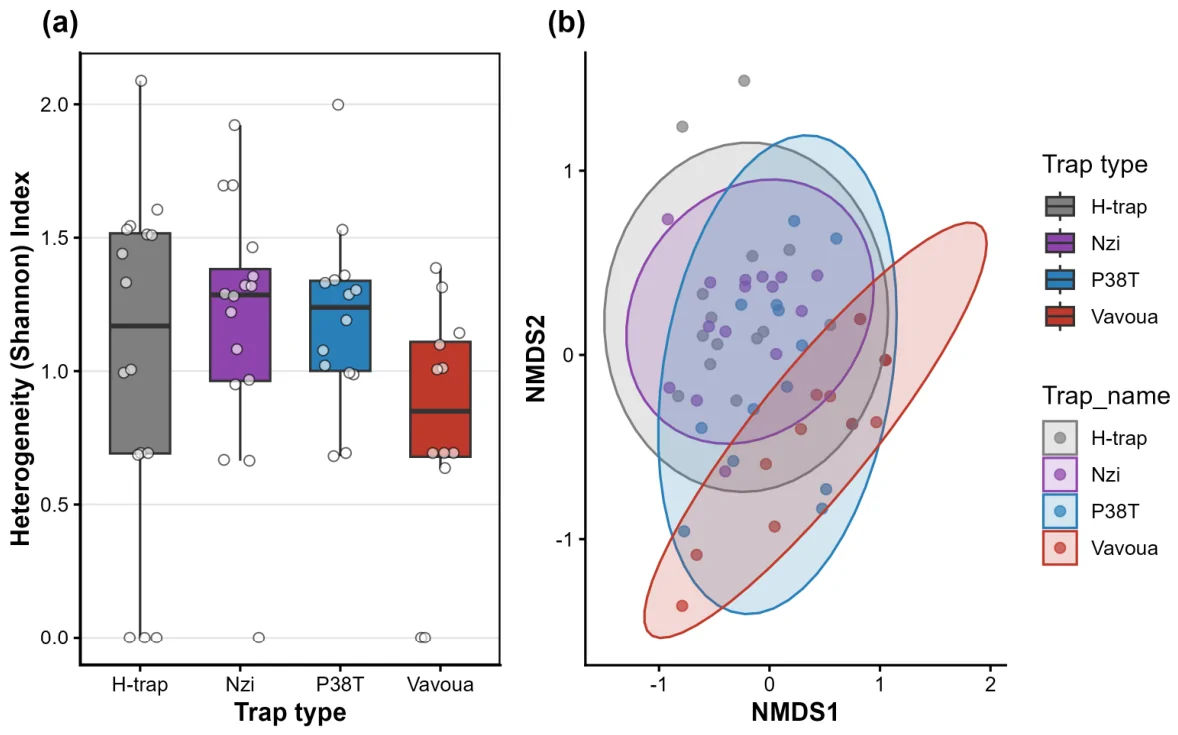
Species surrogate estimations. (**a**) Species heterogeneity/*Alpha* diversity (Shannon) Index within tested traps. (**b**) Community assemblages/*Beta* diversity (NMDS) between tested traps.

The *Alpha* diversity based on the Shannon Index (species richness and evenness) for species caught within a single trap type showed that the Nzi (*H* = 1.286), H-trap (*H* = 1.169) and the P38T BioFlyTrap (*H* = 1.239) had the highest heterogeneity; however, the H-trap had the greatest variation (Figure 5a). Vavoua, on the other hand, demonstrated the lowest Shannon Index (*H* = 0.850), suggesting that it captures a less diverse range of species as compared to the other three traps. Lastly, there was no significant difference between trap type heterogeneity using Shannon diversity.

The *Beta* diversity based on the Bray–Curtis dissimilarities had a NMDS ordination stress value of 0.121 with an acceptable fit, showed that there was visual overlap of community assemblages between the H-trap, Nzi, and the P38T BioFlyTrap, suggesting that these three traps capture similar communities (Figure 5b). Biting fly communities’ composition had significant difference between trap type and sampled sites (*F*_15,45_ = 2.76, *R*^2^ = 0.496, *p* = 0.001), reflecting a shift in PERMANOVA community centroid rather than difference within group variance. Additionally, the H-trap and the P38T BioFlyTrap each had unique species captured. The Vavoua trap showed a more distinct species composition that differs from the other three tested traps.

## 4. Discussion

The current study compared the effectiveness of three odor-baited reference traps (H-trap, Nzi and Vavoua) and the new prototype P38T BioFlyTrap in catching biting flies in northeastern KwaZulu-Natal, South Africa. These findings align with previous studies that have identified the Nzi trap to be efficient in trapping a diverse range of biting species, while the P38T BioFlyTrap moderately outperformed the Vavoua trap, albeit being less versatile as compared to the Nzi and the H-trap, on average daily catch [20,44,45]. The H-trap on the other hand is more specialized for tsetse flies (*Glossina brevipalpis* and *G. austeni*) and Tabanidae in South Africa, which explains its relatively high performance in the study site [19]. The Vavoua has been shown to be more effective in trapping *Stomoxys* spp., which might justify the low catches [45,46].

Species accumulation curves for the H-trap, Nzi, and P38T BioFlyTrap did not approach an asymptote (no plateau) even with 12 biting fly species recorded. This indicated that while the collection was highly representative of Glossinidae [20,29], it captured fewer representatives of Tabanidae [47] and Muscidae (*Stomoxys* spp.) [35]. The curve suggests that extended sampling could yield additional species, although local biting fly diversity was fairly captured within the 16-day sampling period. Consequently, the sampling effort was reasonably sufficient at demonstrating that the P38T BioFlyTrap matches the efficiency of the Nzi and H-traps in capturing diverse biting fly populations [48]. The poor performance of the Vavoua trap across all sampling sites likely stems from insufficient sampling effort [49]. An extended collection period may have yielded higher species diversity. Furthermore, these findings suggest the trap either targets a different demographic of biting insects [45,46] or possesses a lower overall efficiency for capturing diverse fly communities.

*Alpha* diversity revealed that the Nzi and P38T BioFlyTrap do not differ significantly in their overall capacity to sample diverse insect communities. Both traps excelled at capturing a broad spectrum of biting flies, making them ideal for biodiversity mapping [20,46]. In contrast, the Vavoua trap demonstrated significantly low *Alpha* diversity index [45,46]. This low within-trap diversity indicates that the Vavoua design is highly selective, filtering out the broader insect community to focus on a narrow range of target species. The H-trap on the other hand showed a wider range of results including several zero points, suggesting that its performance might be inconsistent with catching a wide range of biting insects as compared to the Nzi and the P38T BioFlyTrap [19]. *Beta* diversity analysis highlighted distinct patterns of community composition across the four trap types. The H-trap, Nzi, and P38T BioFlyTrap showed overlapping communities of biting insects, suggesting that these three traps captured statistically similar multi-species complex; however, no significant difference in their targeted insect communities was observed, meaning they draw from the same local pool of available biting insect species. However, the Vavoua trap diverged entirely, displaying a distinct community composition pattern. This indicates that while the Vavoua trap catches fewer species overall (low *Alpha* diversity), the specific community of species it captures is unique and more selective towards Stomoxyini as compared to the other three trapping devices.

The H-trap was the most specialized in showing consistent catches for Glossinidae and was the only trap with significant records of *Glossina austeni* [19] specifically. These findings are, however, contradictory to those made by Malele et al. [27], who conducted a similar study in three different ecosystems of Tanzania. The authors reported poor performance of the H-trap (0.7%) and Nzi (0.9%) in catching tsetse flies, which was not the case in the current study. This is because the authors did not use bait in the traps as suggested by Kappmeier [19] for the H-trap and Mihok et al. [50] for the Nzi trap. Additionally, their sampled sites were different in vegetation and climate, as well as in the diversity of biting insects from the current study. The Nzi trap had the highest species diversity, and Tabanidae was the most abundant family collected by this trap. Similar observations have been found by [20,44,46,50,51]. The P38T BioFlyTrap was moderately efficient for Stomoxyini, specifically the *Stomoxys* and *Haematobia* species, while being less efficient for Glossinidae and Tabanidae. Similar to the P38T BioFlyTrap, the Vavoua trap showed more preference to Stomoxyini but displayed poor efficiency to Glossinidae and Tabanidae as compared to the P38T BioFlyTrap. The observed preferences in catches from the Vavoua trap are similar to those made by [50] in Kenya, Ref. [46] in Thailand, Refs. [45,52,53] in Cameroon and [51] in Gabon. While abundance varied extensively, the Nzi, H-tap, and P38T BioFlyTrap are equally effective at capturing a diverse range of species (12 total each), whereas the Vavoua is notably less diverse. Additionally, the observed data demonstrate that the number of insects caught varied wildly between individual collection events, whereby some days might have resulted in huge swarms, while others yielded very few.

Lure positioning is a critical factor of trap efficiency. While lures were placed inside the H-trap according to standard protocols, the lack of established guidelines for the other devices required affixing them to the Nzi entrance and the outer borders of the Vavoua and P38T traps. These external placements effectively attract flies to the device vicinity but may fail to stimulate trap entry. Positioning the attractant internally, specifically at the intersection of the blue and black panels within the Vavoua and P38T traps, could optimize catch rates. This internal configuration leverages integrated visual–olfactory cues to guide flies directly into the collection apparatus rather than merely aggregating them around the perimeter. Future studies should systematically evaluate internal placement to standardize sampling protocols.

## 5. Conclusions

While the trap data revealed minor count fluctuations, these variations stem from inherent sampling limitations, fly activity, and community heterogeneity rather than significant population shifts. The H-trap is the most efficient trap for Glossinidae species in South Africa. The Nzi trap had the most diverse Tabanidae species range (nine, versus eight for H-Trap and P38T BioFlyTrap), with Tabanidae being the most dominant family. The Vavoua trap is less diverse and more specific for Muscidae, specifically *Stomoxys* spp. The P38T BioFlyTrap showed moderate performance across all insect families with the highest efficiency for Stomoxyini and Tabanidae than for Glossinidae. Additionally, the P38T BioFlyTrap collected all major biting fly families in high richness and diversity and could be recommended for surveillance and monitoring programs for biting fly communities in vector-disease interventions. However, based on the ecology and environment of northeastern KZN, we therefore suggest that in efforts to have an accurate representation of biting fly communities, more than two types of odor-baited traps should be used and more than one sampling technique should be employed.

## Figures and Tables

**Table 1:** Paired comparison of means (post Tukey test) catches of H, Nzi, P38T and Vavoua traps for 16-day Latin Square rotation experiment.

Trap Types	Estimate	Std Erorr	*Z* Ratio	*p* Value
(H-trap)—Nzi	−0.078	0.161	−0.487	0.962
(H-trap)—P38T	0.449	0.188	2.391	0.079
(H-trap)—Vavoua	1.100	0.219	5.021	<0.000
Nzi—P38T	0.527	0.188	2.807	0.026
Nzi—Vavoua	1.178	0.217	5.425	<0.000
P38T—Vavoua	0.652	0.232	2.809	0.026

**Table 2:** Daily catches indicating total catch, mean and standard deviation as well as trap catch index (*TCI*) of observed biting flies for tested H, Nzi, P38T and Vavoua traps for 16-day Latin Square rotation experiment.

Family	Morphospecies	H-trap	Nzi	P38T	Vavoua	Catch	Mean	Stdev	*TCI*
Glossinidae	*Glossina brevipalpis*	10.250	4.813	0.625	0.063	15.750	3.938	±4.712	0.175
	*Glossina austeni*	0.375	0.000	0.000	0.000	0.375	0.094	±0.188	0.004
Muscidae	*Stomoxys niger niger*	2.438	2.500	5.250	5.750	15.938	3.984	±1.762	0.177
	*Stomoxys calitrans*	0.750	1.313	0.813	1.500	4.375	1.094	±0.370	0.049
	*Haematoboia sp.*	0.000	0.000	0.563	0.000	0.563	0.141	±0.281	0.006
Tabanidae	*Ancala Africana*	0.188	0.750	0.000	0.000	0.938	0.234	±0.355	0.010
	*Chrysops sp.*	0.125	0.125	1.125	1.313	2.688	0.672	±0.636	0.030
	*Philoliche aethiopica*	0.125	0.000	0.000	0.000	0.125	0.031	±0.063	0.001
	*Haematopota sp.*	0.000	0.000	0.563	0.125	0.688	0.172	±0.267	0.008
	*Haematopota pardalina*	0.000	0.000	0.063	0.000	0.063	0.016	±0.031	0.001
	*Hybomitra sp.*	0.063	0.063	0.000	0.063	0.188	0.047	±0.031	0.002
	*Tabanocella sp.*	0.000	0.000	0.250	0.000	0.250	0.063	±0.125	0.003
	*Tabanus insignius*	0.063	0.063	0.125	0.000	0.250	0.063	±0.051	0.003
	*Tabanus ustus*	2.938	5.813	1.375	0.188	10.313	2.578	±2.433	0.115
	*Tabanus par*	10.563	19.750	3.625	0.938	34.875	8.719	±8.398	0.388
	*Tabanus gratus*	0.000	0.313	0.000	0.000	0.313	0.078	±0.156	0.003
	*Tabanus taeniola*	0.438	0.875	0.750	0.000	2.063	0.516	±0.390	0.023
	*Tabanus zuluenisis*	0.000	0.188	0.000	0.000	0.188	0.047	±0.094	0.002

## Data Availability

Raw data from each trap and number of insect species collected are provided in Supplementary Table S1.

## Supplementary Materials

The following supporting information can be downloaded at: https://www.mdpi.com/article/10.3390/insects17090912/s1. Table S1: Raw data of all biting insects collected.

## Abbreviations

The following abbreviations are used in this manuscript:

AAT	Animal African Trypanosomosis
COMBAT	Controlling and Minimizing the Burden of Animal Trypanosomosis
GLMM	Generalized Linear Mixed Model
NMDS	Non-metric Multidimensional Scaling Ordination
LSD	Latin Square Design
