# Supplementary material for: Evaluating the Efficiency of Baited BioFlyTrap P38T, H-Trap, and Nzi and Vavoua Traps Towards Biting Flies of Glossinidae, Tabanidae and Muscidae (Stomoxyini) Families in Northeastern KwaZulu-Natal
Source: Insects. 2026 Sep 1;17(9):912. doi: 10.3390/insects17090912 (PMC13607364; doi:10.3390/insects17090912)
Supplement: Supplementary file 1 [file insects-17-00912-s001.zip › insects-4439367-supplementary.pdf]

Table S1: Raw data of all biting insects collected.

Survey_ID	Site_ID	Latitude	Longitude	Sart_date	End_date	Trapping_days	Trap_name	Rotation	Family	Species_ID	Counts
1	Site A	-28,352032	32,431357	05-Nov-25	6-Nov-25	1	H-trap	1	Glossinidae	*Glossina_brevipalpis*	31
2	Site A	-28,352032	32,431357	05-Nov-25	6-Nov-25	1	H-trap	1	Tabanidae	*Tabanus_par*	9
3	Site A	-28,352032	32,431357	05-Nov-25	6-Nov-25	1	H-trap	1	Tabanidae	*Tabanus_taeniola*	4
4	Site A	-28,352032	32,431357	05-Nov-25	6-Nov-25	1	H-trap	1	Muscidae	*Stomoxys_niger_niger*	4
5	Site C	-28,35065	32,431543	05-Nov-25	6-Nov-25	1	Nzi	1	Tabanidae	*Tabanus_par*	1
6	Site D	-28,349917	32,431687	05-Nov-25	6-Nov-25	1	P38T	1	Glossinidae	*Glossina_brevipalpis*	2
7	Site D	-28,349917	32,431687	05-Nov-25	6-Nov-25	1	P38T	1	Tabanidae	*Tabanus_par*	1
8	Site D	-28,349917	32,431687	05-Nov-25	6-Nov-25	1	P38T	1	Tabanidae	*Tabanus_taeniola*	2
9	Site A	-28,352032	32,431357	06-Nov-25	7-Nov-25	1	Vavoua	2	Tabanidae	*Haematopota_* sp.1	2
10	Site A	-28,352032	32,431357	06-Nov-25	7-Nov-25	1	Vavoua	2	Tabanidae	*Chrysops_* sp.1	15
11	Site A	-28,352032	32,431357	06-Nov-25	7-Nov-25	1	Vavoua	2	Muscidae	*Stomoxys_calcitrans*	20
12	Site A	-28,352032	32,431357	06-Nov-25	7-Nov-25	1	Vavoua	2	Muscidae	*Stomoxys_niger_niger*	35
13	Site B	-28,351067	32,431543	06-Nov-25	7-Nov-25	1	Nzi	2	Glossinidae	*Glossina_brevipalpis*	1
14	Site B	-28,351067	32,431543	06-Nov-25	7-Nov-25	1	Nzi	2	Tabanidae	*Tabanus_par*	2
15	Site C	-28,35065	32,431543	06-Nov-25	7-Nov-25	1	P38T	2	Tabanidae	*Tabanus_par*	2
16	Site C	-28,35065	32,431543	06-Nov-25	7-Nov-25	1	P38T	2	Tabanidae	*Tabanus_taeniola*	1
17	Site C	-28,35065	32,431543	06-Nov-25	7-Nov-25	1	P38T	2	Tabanidae	*Haematopota_pardalina*	1
18	Site C	-28,35065	32,431543	06-Nov-25	7-Nov-25	1	P38T	2	Tabanidae	*Chrysops_* sp.1	3
19	Site D	-28,349917	32,431687	06-Nov-25	7-Nov-25	1	H-trap	2	Glossinidae	*Glossina_brevipalpis*	8
20	Site D	-28,349917	32,431687	06-Nov-25	7-Nov-25	1	H-trap	2	Tabanidae	*Tabanus_par*	4
21	Site D	-28,349917	32,431687	06-Nov-25	7-Nov-25	1	H-trap	2	Tabanidae	*Tabanus_taeniola*	1
22	Site A	-28,352032	32,431357	07-Nov-25	9-Nov-25	2	Nzi	3	Glossinidae	*Glossina_brevipalpis*	16
23	Site A	-28,352032	32,431357	07-Nov-25	9-Nov-25	2	Nzi	3	Tabanidae	*Tabanus_par*	141
24	Site A	-28,352032	32,431357	07-Nov-25	9-Nov-25	2	Nzi	3	Tabanidae	*Tabanus_insignius*	1
25	Site A	-28,352032	32,431357	07-Nov-25	9-Nov-25	2	Nzi	3	Tabanidae	*Tabanus_ustus*	53
26	Site A	-28,352032	32,431357	07-Nov-25	9-Nov-25	2	Nzi	3	Tabanidae	*Hybomitra_* sp.1	1
27	Site A	-28,352032	32,431357	07-Nov-25	9-Nov-25	2	Nzi	3	Tabanidae	*Ancala_africana*	7
28	Site A	-28,352032	32,431357	07-Nov-25	9-Nov-25	2	Nzi	3	Muscidae	*Stomoxys_calcitrans*	10
29	Site A	-28,352032	32,431357	07-Nov-25	9-Nov-25	2	Nzi	3	Muscidae	*Stomoxys_niger_niger*	15
30	Site B	-28,351067	32,431543	07-Nov-25	9-Nov-25	2	P38T	3	Tabanidae	*Tabanus_par*	6
31	Site B	-28,351067	32,431543	07-Nov-25	9-Nov-25	2	P38T	3	Tabanidae	*Tabanus_taeniola*	1
32	Site B	-28,351067	32,431543	07-Nov-25	9-Nov-25	2	P38T	3	Tabanidae	*Tabanus_ustus*	4
33	Site C	-28,35065	32,431543	07-Nov-25	9-Nov-25	2	H-trap	3	Glossinidae	*Glossina_brevipalpis*	16
34	Site C	-28,35065	32,431543	07-Nov-25	9-Nov-25	2	H-trap	3	Glossinidae	*Glossina_austeni*	1
35	Site C	-28,35065	32,431543	07-Nov-25	9-Nov-25	2	H-trap	3	Tabanidae	*Tabanus_par*	18
36	Site C	-28,35065	32,431543	07-Nov-25	9-Nov-25	2	H-trap	3	Tabanidae	*Tabanus_ustus*	20
37	Site C	-28,35065	32,431543	07-Nov-25	9-Nov-25	2	H-trap	3	Tabanidae	*Chrysops_* sp.1	1

38	Site D	-28,349917	32,431687	07-Nov-25	9-Nov-25	2	Vavoua	3	Glossinidae	*Glossina_brevipalpis*	1
39	Site D	-28,349917	32,431687	07-Nov-25	9-Nov-25	2	Vavoua	3	Tabanidae	*Tabanus_par*	1
40	Site D	-28,349917	32,431687	07-Nov-25	9-Nov-25	2	Vavoua	3	Tabanidae	*Chrysops_* sp.1	1
41	Site D	-28,349917	32,431687	07-Nov-25	9-Nov-25	2	Vavoua	3	Muscidae	*Stomoxys_niger_niger*	1
42	Site A	-28,352032	32,431357	09-Nov-25	10-Nov-25	1	P38T	4	Glossinidae	*Glossina_brevipalpis*	2
43	Site A	-28,352032	32,431357	09-Nov-25	10-Nov-25	1	P38T	4	Tabanidae	*Tabanus_par*	7
44	Site A	-28,352032	32,431357	09-Nov-25	10-Nov-25	1	P38T	4	Tabanidae	*Tabanus_taeniola*	2
45	Site A	-28,352032	32,431357	09-Nov-25	10-Nov-25	1	P38T	4	Tabanidae	*Tabanus_ustus*	5
46	Site A	-28,352032	32,431357	09-Nov-25	10-Nov-25	1	P38T	4	Tabanidae	*Tabanocella_* sp.1	4
47	Site A	-28,352032	32,431357	09-Nov-25	10-Nov-25	1	P38T	4	Tabanidae	*Chrysops_* sp.1	10
48	Site A	-28,352032	32,431357	09-Nov-25	10-Nov-25	1	P38T	4	Muscidae	*Stomoxys_calcitrans*	2
49	Site A	-28,352032	32,431357	09-Nov-25	10-Nov-25	1	P38T	4	Muscidae	*Stomoxys_niger_niger*	30
50	Site B	-28,351067	32,431543	09-Nov-25	10-Nov-25	1	H-trap	4	Glossinidae	*Glossina_brevipalpis*	3
51	Site B	-28,351067	32,431543	09-Nov-25	10-Nov-25	1	H-trap	4	Glossinidae	*Glossina_austeni*	3
52	Site C	-28,35065	32,431543	09-Nov-25	10-Nov-25	1	Vavoua	4	Tabanidae	*Tabanus_par*	1
53	Site C	-28,35065	32,431543	09-Nov-25	10-Nov-25	1	Vavoua	4	Tabanidae	*Tabanus_ustus*	1
54	Site C	-28,35065	32,431543	09-Nov-25	10-Nov-25	1	Vavoua	4	Muscidae	*Stomoxys_niger_niger*	1
55	Site D	-28,349917	32,431687	09-Nov-25	10-Nov-25	1	Nzi	4	Glossinidae	*Glossina_brevipalpis*	7
56	Site D	-28,349917	32,431687	09-Nov-25	10-Nov-25	1	Nzi	4	Tabanidae	*Tabanus_gratus*	5
57	Site D	-28,349917	32,431687	09-Nov-25	10-Nov-25	1	Nzi	4	Tabanidae	*Tabanus_taeniola*	1
58	Site D	-28,349917	32,431687	09-Nov-25	10-Nov-25	1	Nzi	4	Tabanidae	*Tabanus_ustus*	3
59	Site A	-28,352032	32,431357	10-Nov-25	11-Nov-25	1	H-trap	5	Glossinidae	*Glossina_brevipalpis*	37
60	Site A	-28,352032	32,431357	10-Nov-25	11-Nov-25	1	H-trap	5	Tabanidae	*Tabanus_par*	48
61	Site A	-28,352032	32,431357	10-Nov-25	11-Nov-25	1	H-trap	5	Tabanidae	*Tabanus_insignius*	1
62	Site A	-28,352032	32,431357	10-Nov-25	11-Nov-25	1	H-trap	5	Tabanidae	*Tabanus_ustus*	11
63	Site A	-28,352032	32,431357	10-Nov-25	11-Nov-25	1	H-trap	5	Tabanidae	*Hybomitra_* sp.1	1
64	Site A	-28,352032	32,431357	10-Nov-25	11-Nov-25	1	H-trap	5	Tabanidae	*Ancala_africana*	2
65	Site A	-28,352032	32,431357	10-Nov-25	11-Nov-25	1	H-trap	5	Tabanidae	*Philoliche_aethiopica*	1
66	Site A	-28,352032	32,431357	10-Nov-25	11-Nov-25	1	H-trap	5	Tabanidae	*Chrysops_* sp.1	1
67	Site A	-28,352032	32,431357	10-Nov-25	11-Nov-25	1	H-trap	5	Muscidae	*Stomoxys_calcitrans*	9
68	Site A	-28,352032	32,431357	10-Nov-25	11-Nov-25	1	H-trap	5	Muscidae	*Stomoxys_niger_niger*	19
69	Site B	-28,351067	32,431543	10-Nov-25	11-Nov-25	1	Vavoua	5	Tabanidae	*Tabanus_par*	1
70	Site B	-28,351067	32,431543	10-Nov-25	11-Nov-25	1	Vavoua	5	Tabanidae	*Tabanus_ustus*	1
71	Site C	-28,35065	32,431543	10-Nov-25	11-Nov-25	1	Nzi	5	Glossinidae	*Glossina_brevipalpis*	2
72	Site C	-28,35065	32,431543	10-Nov-25	11-Nov-25	1	Nzi	5	Tabanidae	*Tabanus_par*	23
73	Site C	-28,35065	32,431543	10-Nov-25	11-Nov-25	1	Nzi	5	Tabanidae	*Tabanus_ustus*	8
74	Site C	-28,35065	32,431543	10-Nov-25	11-Nov-25	1	Nzi	5	Tabanidae	*Ancala_africana*	1
75	Site C	-28,35065	32,431543	10-Nov-25	11-Nov-25	1	Nzi	5	Muscidae	*Stomoxys_niger_niger*	2

76	Site D	-28,349917	32,431687	10-Nov-25	11-Nov-25	1	P38T	5	Tabanidae	*Tabanus_par*	8
77	Site D	-28,349917	32,431687	10-Nov-25	11-Nov-25	1	P38T	5	Tabanidae	*Tabanus_ustus*	4
78	Site A	-28,352032	32,431357	11-Nov-25	12-Nov-25	1	Vavoua	6	Tabanidae	*Tabanus_ustus*	1
79	Site A	-28,352032	32,431357	11-Nov-25	12-Nov-25	1	Vavoua	6	Tabanidae	*Chrysops_* sp.1	1
80	Site A	-28,352032	32,431357	11-Nov-25	12-Nov-25	1	Vavoua	6	Muscidae	*Stomoxys_calcitrans*	4
81	Site A	-28,352032	32,431357	11-Nov-25	12-Nov-25	1	Vavoua	6	Muscidae	*Stomoxys_niger_niger*	38
82	Site B	-28,351067	32,431543	11-Nov-25	12-Nov-25	1	Nzi	6	Glossinidae	*Glossina_brevipalpis*	4
83	Site B	-28,351067	32,431543	11-Nov-25	12-Nov-25	1	Nzi	6	Tabanidae	*Tabanus_par*	16
84	Site B	-28,351067	32,431543	11-Nov-25	12-Nov-25	1	Nzi	6	Tabanidae	*Tabanus_taeniola*	12
85	Site B	-28,351067	32,431543	11-Nov-25	12-Nov-25	1	Nzi	6	Tabanidae	*Tabanus_zuluensis*	1
86	Site B	-28,351067	32,431543	11-Nov-25	12-Nov-25	1	Nzi	6	Tabanidae	*Tabanus_ustus*	8
87	Site B	-28,351067	32,431543	11-Nov-25	12-Nov-25	1	Nzi	6	Muscidae	*Stomoxys_niger_niger*	2
88	Site C	-28,35065	32,431543	11-Nov-25	12-Nov-25	1	P38T	6	Glossinidae	*Glossina_brevipalpis*	4
89	Site C	-28,35065	32,431543	11-Nov-25	12-Nov-25	1	P38T	6	Tabanidae	*Tabanus_par*	5
90	Site C	-28,35065	32,431543	11-Nov-25	12-Nov-25	1	P38T	6	Tabanidae	*Tabanus_ustus*	5
91	Site C	-28,35065	32,431543	11-Nov-25	12-Nov-25	1	P38T	6	Tabanidae	*Chrysops_* sp.1	1
92	Site D	-28,349917	32,431687	11-Nov-25	12-Nov-25	1	H-trap	6	Glossinidae	*Glossina_brevipalpis*	15
93	Site D	-28,349917	32,431687	11-Nov-25	12-Nov-25	1	H-trap	6	Glossinidae	*Glossina_austeni*	2
94	Site D	-28,349917	32,431687	11-Nov-25	12-Nov-25	1	H-trap	6	Tabanidae	*Tabanus_par*	15
95	Site D	-28,349917	32,431687	11-Nov-25	12-Nov-25	1	H-trap	6	Tabanidae	*Tabanus_ustus*	5
96	Site D	-28,349917	32,431687	11-Nov-25	12-Nov-25	1	H-trap	6	Muscidae	*Stomoxys_niger_niger*	2
97	Site A	-28,352032	32,431357	12-Nov-25	13-Nov-25	1	Nzi	7	Glossinidae	*Glossina_brevipalpis*	20
98	Site A	-28,352032	32,431357	12-Nov-25	13-Nov-25	1	Nzi	7	Tabanidae	*Tabanus_par*	43
99	Site A	-28,352032	32,431357	12-Nov-25	13-Nov-25	1	Nzi	7	Tabanidae	*Tabanus_ustus*	2
100	Site A	-28,352032	32,431357	12-Nov-25	13-Nov-25	1	Nzi	7	Tabanidae	*Chrysops_* sp.1	2
101	Site A	-28,352032	32,431357	12-Nov-25	13-Nov-25	1	Nzi	7	Muscidae	*Stomoxys_calcitrans*	7
102	Site A	-28,352032	32,431357	12-Nov-25	13-Nov-25	1	Nzi	7	Muscidae	*Stomoxys_niger_niger*	10
103	Site B	-28,351067	32,431543	12-Nov-25	13-Nov-25	1	P38T	7	Tabanidae	*Tabanus_par*	1
104	Site B	-28,351067	32,431543	12-Nov-25	13-Nov-25	1	P38T	7	Muscidae	*Stomoxys_niger_niger*	1
105	Site C	-28,35065	32,431543	12-Nov-25	13-Nov-25	1	H-trap	7	Glossinidae	*Glossina_brevipalpis*	3
106	Site C	-28,35065	32,431543	12-Nov-25	13-Nov-25	1	H-trap	7	Tabanidae	*Tabanus_par*	9
107	Site C	-28,35065	32,431543	12-Nov-25	13-Nov-25	1	H-trap	7	Muscidae	*Stomoxys_niger_niger*	1
108	Site D	-28,349917	32,431687	12-Nov-25	13-Nov-25	1	Vavoua	7	Tabanidae	*Tabanus_par*	1
109	Site D	-28,349917	32,431687	12-Nov-25	13-Nov-25	1	Vavoua	7	Tabanidae	*Chrysops_* sp.1	1
110	Site A	-28,352032	32,431357	13-Nov-25	14-Nov-25	1	P38T	8	Glossinidae	*Glossina_brevipalpis*	1
111	Site A	-28,352032	32,431357	13-Nov-25	14-Nov-25	1	P38T	8	Tabanidae	*Chrysops_* sp.1	2
112	Site A	-28,352032	32,431357	13-Nov-25	14-Nov-25	1	P38T	8	Muscidae	*Stomoxys_calcitrans*	4
113	Site A	-28,352032	32,431357	13-Nov-25	14-Nov-25	1	P38T	8	Muscidae	*Stomoxys_niger_niger*	40

114	Site B	-28,351067	32,431543	13-Nov-25	14-Nov-25	1	H-trap	8	Tabanidae	*Tabanus_par*	1
115	Site C	-28,35065	32,431543	13-Nov-25	14-Nov-25	1	Vavoua	8	Tabanidae	*Tabanus_par*	1
116	Site C	-28,35065	32,431543	13-Nov-25	14-Nov-25	1	Vavoua	8	Muscidae	*Stomoxys_niger_niger*	1
117	Site D	-28,349917	32,431687	13-Nov-25	14-Nov-25	1	Nzi	8	Glossinidae	*Glossina_brevipalpis*	3
118	Site D	-28,349917	32,431687	13-Nov-25	14-Nov-25	1	Nzi	8	Tabanidae	*Tabanus_par*	11
119	Site D	-28,349917	32,431687	13-Nov-25	14-Nov-25	1	Nzi	8	Tabanidae	*Tabanus_ustus*	2
120	Site D	-28,349917	32,431687	13-Nov-25	14-Nov-25	1	Nzi	8	Tabanidae	*Ancala_africana*	1
121	Site A	-28,352032	32,431357	14-Nov-25	15-Nov-25	1	H-trap	9	Glossinidae	*Glossina_brevipalpis*	17
122	Site A	-28,352032	32,431357	14-Nov-25	15-Nov-25	1	H-trap	9	Tabanidae	*Tabanus_par*	14
123	Site A	-28,352032	32,431357	14-Nov-25	15-Nov-25	1	H-trap	9	Tabanidae	*Tabanus_taeniola*	1
124	Site A	-28,352032	32,431357	14-Nov-25	15-Nov-25	1	H-trap	9	Tabanidae	*Tabanus_ustus*	4
125	Site A	-28,352032	32,431357	14-Nov-25	15-Nov-25	1	H-trap	9	Muscidae	*Stomoxys_niger_niger*	4
126	Site B	-28,351067	32,431543	14-Nov-25	15-Nov-25	1	Vavoua	9	Tabanidae	*Tabanus_par*	3
127	Site B	-28,351067	32,431543	14-Nov-25	15-Nov-25	1	Vavoua	9	Muscidae	*Stomoxys_niger_niger*	1
128	Site C	-28,35065	32,431543	14-Nov-25	15-Nov-25	1	Nzi	9	Glossinidae	*Glossina_brevipalpis*	2
129	Site C	-28,35065	32,431543	14-Nov-25	15-Nov-25	1	Nzi	9	Tabanidae	*Tabanus_par*	4
130	Site C	-28,35065	32,431543	14-Nov-25	15-Nov-25	1	Nzi	9	Tabanidae	*Tabanus_taeniola*	1
131	Site C	-28,35065	32,431543	14-Nov-25	15-Nov-25	1	Nzi	9	Tabanidae	*Tabanus_ustus*	1
132	Site D	-28,349917	32,431687	14-Nov-25	15-Nov-25	1	P38T	9	Tabanidae	*Tabanus_par*	3
133	Site D	-28,349917	32,431687	14-Nov-25	15-Nov-25	1	P38T	9	Tabanidae	*Tabanus_ustus*	1
134	Site D	-28,349917	32,431687	14-Nov-25	15-Nov-25	1	P38T	9	Muscidae	*Haematoboia_* sp.1	9
135	Site A	-28,352032	32,431357	15-Nov-25	16-Nov-25	1	Vavoua	10	Tabanidae	*Tabanus_par*	1
136	Site A	-28,352032	32,431357	15-Nov-25	16-Nov-25	1	Vavoua	10	Tabanidae	*Chrysops_* sp.1	3
137	Site A	-28,352032	32,431357	15-Nov-25	16-Nov-25	1	Vavoua	10	Muscidae	*Stomoxys_niger_niger*	7
138	Site B	-28,351067	32,431543	15-Nov-25	16-Nov-25	1	Nzi	10	Glossinidae	*Glossina_brevipalpis*	2
139	Site B	-28,351067	32,431543	15-Nov-25	16-Nov-25	1	Nzi	10	Tabanidae	*Tabanus_par*	5
140	Site C	-28,35065	32,431543	15-Nov-25	16-Nov-25	1	P38T	10	Tabanidae	*Tabanus_par*	8
141	Site C	-28,35065	32,431543	15-Nov-25	16-Nov-25	1	P38T	10	Tabanidae	*Tabanus_taeniola*	2
142	Site C	-28,35065	32,431543	15-Nov-25	16-Nov-25	1	P38T	10	Muscidae	*Stomoxys_niger_niger*	1
143	Site D	-28,349917	32,431687	15-Nov-25	16-Nov-25	1	H-trap	10	Tabanidae	*Tabanus_par*	4
144	Site A	-28,352032	32,431357	16-Nov-25	17-Nov-25	1	Nzi	11	Glossinidae	*Glossina_brevipalpis*	2
145	Site A	-28,352032	32,431357	16-Nov-25	17-Nov-25	1	Nzi	11	Tabanidae	*Tabanus_par*	1
146	Site A	-28,352032	32,431357	16-Nov-25	17-Nov-25	1	Nzi	11	Muscidae	*Stomoxys_calcitrans*	4
147	Site A	-28,352032	32,431357	16-Nov-25	17-Nov-25	1	Nzi	11	Muscidae	*Stomoxys_niger_niger*	10
148	Site C	-28,351067	32,431543	16-Nov-25	17-Nov-25	1	H-trap	11	Glossinidae	*Glossina_brevipalpis*	1
149	Site A	-28,352032	32,431357	17-Nov-25	18-Nov-25	1	P38T	12	Tabanidae	*Tabanus_par*	4
150	Site A	-28,352032	32,431357	17-Nov-25	18-Nov-25	1	P38T	12	Tabanidae	*Tabanus_ustus*	2
151	Site A	-28,352032	32,431357	17-Nov-25	18-Nov-25	1	P38T	12	Tabanidae	*Chrysops_* sp.1	1

152	Site A	-28,352032	32,431357	17-Nov-25	18-Nov-25	1	P38T	12	Muscidae	*Stomoxys_calcitrans*	6
153	Site A	-28,352032	32,431357	17-Nov-25	18-Nov-25	1	P38T	12	Muscidae	*Stomoxys_niger_niger*	10
154	Site B	-28,351067	32,431543	17-Nov-25	18-Nov-25	1	H-trap	12	Tabanidae	*Tabanus_ustus*	1
155	Site B	-28,351067	32,431543	17-Nov-25	18-Nov-25	1	H-trap	12	Tabanidae	*Tabanus_par*	1
156	Site D	-28,349917	32,431687	17-Nov-25	18-Nov-25	1	Nzi	12	Tabanidae	*Tabanus_par*	6
157	Site D	-28,349917	32,431687	17-Nov-25	18-Nov-25	1	Nzi	12	Tabanidae	*Tabanus_ustus*	1
158	Site D	-28,349917	32,431687	17-Nov-25	18-Nov-25	1	Nzi	12	Tabanidae	*Ancala_africana*	1
159	Site A	-28,352032	32,431357	18-Nov-25	19-Nov-25	1	H-trap	13	Glossinidae	*Glossina_brevipalpis*	27
160	Site A	-28,352032	32,431357	18-Nov-25	19-Nov-25	1	H-trap	13	Tabanidae	*Tabanus_par*	32
161	Site A	-28,352032	32,431357	18-Nov-25	19-Nov-25	1	H-trap	13	Tabanidae	*Tabanus_taeniola*	1
162	Site A	-28,352032	32,431357	18-Nov-25	19-Nov-25	1	H-trap	13	Tabanidae	*Tabanus_ustus*	4
163	Site A	-28,352032	32,431357	18-Nov-25	19-Nov-25	1	H-trap	13	Tabanidae	*Philoliche_aethiopica*	1
164	Site A	-28,352032	32,431357	18-Nov-25	19-Nov-25	1	H-trap	13	Muscidae	*Stomoxys_niger_niger*	3
165	Site B	-28,351067	32,431543	18-Nov-25	19-Nov-25	1	Vavoua	13	Tabanidae	*Tabanus_par*	1
166	Site C	-28,35065	32,431543	18-Nov-25	19-Nov-25	1	Nzi	13	Glossinidae	*Glossina_brevipalpis*	2
167	Site C	-28,35065	32,431543	18-Nov-25	19-Nov-25	1	Nzi	13	Tabanidae	*Tabanus_par*	6
168	Site C	-28,35065	32,431543	18-Nov-25	19-Nov-25	1	Nzi	13	Tabanidae	*Tabanus_ustus*	4
169	Site C	-28,35065	32,431543	18-Nov-25	19-Nov-25	1	Nzi	13	Tabanidae	*Ancala_africana*	2
170	Site D	-28,349917	32,431687	18-Nov-25	19-Nov-25	1	P38T	13	Tabanidae	*Tabanus_par*	4
171	Site D	-28,349917	32,431687	18-Nov-25	19-Nov-25	1	P38T	13	Tabanidae	*Tabanus_taeniola*	1
172	Site D	-28,349917	32,431687	18-Nov-25	19-Nov-25	1	P38T	13	Tabanidae	*Tabanus_ustus*	1
173	Site D	-28,349917	32,431687	18-Nov-25	19-Nov-25	1	P38T	13	Tabanidae	*Chrysops_* sp.1	1
174	Site A	-28,352032	32,431357	19-Nov-25	20-Nov-25	1	Vavoua	14	Tabanidae	*Tabanus_par*	4
175	Site A	-28,352032	32,431357	19-Nov-25	20-Nov-25	1	Vavoua	14	Tabanidae	*Hybomitra_* sp.1	1
176	Site A	-28,352032	32,431357	19-Nov-25	20-Nov-25	1	Vavoua	14	Muscidae	*Stomoxys_niger_niger*	8
177	Site B	-28,351067	32,431543	19-Nov-25	20-Nov-25	1	Nzi	14	Glossinidae	*Glossina_brevipalpis*	1
178	Site B	-28,351067	32,431543	19-Nov-25	20-Nov-25	1	Nzi	14	Tabanidae	*Tabanus_par*	7
179	Site B	-28,351067	32,431543	19-Nov-25	20-Nov-25	1	Nzi	14	Muscidae	*Stomoxys_niger_niger*	1
180	Site C	-28,35065	32,431543	19-Nov-25	20-Nov-25	1	P38T	14	Tabanidae	*Tabanus_par*	2
181	Site C	-28,35065	32,431543	19-Nov-25	20-Nov-25	1	P38T	14	Tabanidae	*Tabanus_taeniola*	3
182	Site C	-28,35065	32,431543	19-Nov-25	20-Nov-25	1	P38T	14	Tabanidae	*Tabanus_insignius*	2
183	Site C	-28,35065	32,431543	19-Nov-25	20-Nov-25	1	P38T	14	Muscidae	*Stomoxys_calcitrans*	1
184	Site D	-28,349917	32,431687	19-Nov-25	20-Nov-25	1	H-trap	14	Glossinidae	*Glossina_brevipalpis*	1
185	Site D	-28,349917	32,431687	19-Nov-25	20-Nov-25	1	H-trap	14	Tabanidae	*Tabanus_par*	8
186	Site D	-28,349917	32,431687	19-Nov-25	20-Nov-25	1	H-trap	14	Tabanidae	*Tabanus_ustus*	1
187	Site D	-28,349917	32,431687	19-Nov-25	20-Nov-25	1	H-trap	14	Muscidae	*Stomoxys_calcitrans*	3
188	Site D	-28,349917	32,431687	19-Nov-25	20-Nov-25	1	H-trap	14	Muscidae	*Stomoxys_niger_niger*	5
189	Site A	-28,352032	32,431357	19-Nov-25	21-Nov-25	2	Nzi	15	Glossinidae	*Glossina_brevipalpis*	14

190	Site A	-28,352032	32,431357	20-Nov-25	21-Nov-25	1	Nzi	15	Tabanidae	*Tabanus_par*	38
191	Site A	-28,352032	32,431357	20-Nov-25	21-Nov-25	1	Nzi	15	Tabanidae	*Tabanus_ustus*	10
192	Site C	-28,35065	32,431543	20-Nov-25	21-Nov-25	1	H-trap	15	Glossinidae	*Glossina_brevipalpis*	2
193	Site C	-28,35065	32,431543	20-Nov-25	21-Nov-25	1	H-trap	15	Tabanidae	*Tabanus_par*	4
194	Site C	-28,35065	32,431543	20-Nov-25	21-Nov-25	1	H-trap	15	Tabanidae	*Tabanus_ustus*	1
195	Site C	-28,35065	32,431543	20-Nov-25	21-Nov-25	1	H-trap	15	Tabanidae	*Ancala_africana*	1
196	Site C	-28,35065	32,431543	20-Nov-25	21-Nov-25	1	H-trap	15	Muscidae	*Stomoxys_niger_niger*	1
197	Site A	-28,352032	32,431357	21-Nov-25	22-Nov-25	1	P38T	16	Glossinidae	*Glossina_brevipalpis*	1
198	Site A	-28,352032	32,431357	21-Nov-25	22-Nov-25	1	P38T	16	Tabanidae	*Tabanus_par*	7
199	Site A	-28,352032	32,431357	21-Nov-25	22-Nov-25	1	P38T	16	Tabanidae	*Haematopota_* sp.1	9
200	Site A	-28,352032	32,431357	21-Nov-25	22-Nov-25	1	P38T	16	Muscidae	*Stomoxys_niger_niger*	2
201	Site B	-28,351067	32,431543	21-Nov-25	22-Nov-25	1	H-trap	16	Glossinidae	*Glossina_brevipalpis*	3
202	Site B	-28,351067	32,431543	21-Nov-25	22-Nov-25	1	H-trap	16	Tabanidae	*Tabanus_par*	2
203	Site C	-28,35065	32,431543	21-Nov-25	22-Nov-25	1	Vavoua	16	Tabanidae	*Tabanus_par*	1
204	Site D	-28,349917	32,431687	21-Nov-25	22-Nov-25	1	Nzi	16	Glossinidae	*Glossina_brevipalpis*	1
205	Site D	-28,349917	32,431687	21-Nov-25	22-Nov-25	1	Nzi	16	Tabanidae	*Tabanus_par*	12
206	Site D	-28,349917	32,431687	21-Nov-25	22-Nov-25	1	Nzi	16	Tabanidae	*Tabanus_zuluensis*	2
207	Site D	-28,349917	32,431687	21-Nov-25	22-Nov-25	1	Nzi	16	Tabanidae	*Tabanus_ustus*	1
											1439
